# Polymorphisms in the type A *blaZ* gene as determinants of the cefazolin inoculum effect in *Staphylococcus aureus*

**DOI:** 10.1128/aac.01106-24

**Published:** 2024-12-10

**Authors:** Shinwon Lee, Sohee Park, Jeong Nam Kim, Soon Ok Lee, Jeong Eun Lee, Sun Hee Lee

**Affiliations:** 1 Department of Internal Medicine, Pusan National University School of Medicine and Medical Research Institute, Pusan National University Hospital 220312 , Busan, Republic of Korea; 2 Department of Microbiology, College of Natural Sciences, Pusan National University 124640 , Busan, Republic of Korea; The Peter Doherty Institute for Infection and Immunity, Melbourne, Australia

**Keywords:** *Staphylococcus aureus*, cefazolin, inoculum effect, beta-lactamase, polymorphism

## Abstract

Plasmids containing the wild-type (WT) or mutant type A *blaZ* (*blaZ*_A_) gene (mutations at codons 226, 229, or both 226 and 229) were constructed and transformed into *Staphylococcus aureus* RN4220, yielding the strains WT-blaZ, M226-blaZ, M229-blaZ, and MB-blaZ. The high-inoculum cefazolin MIC was significantly lower in MB-blaZ and M226-blaZ than in WT-blaZ but not in M229-blaZ, suggesting that the single nucleotide polymorphism at codon 226 in *blaZ*_A_ contributes to the cefazolin inoculum effect.

## INTRODUCTION

Cefazolin is widely used to treat methicillin-susceptible *Staphylococcus aureus* (MSSA) infections (1–4). However, certain MSSA strains exhibit a cefazolin inoculum effect (CIE), which can be associated with treatment failure in clinical settings (5–8). The inoculum effect against β-lactam antibiotics is closely related to the specific type of β-lactamase (Bla), a β-lactam-hydrolyzing enzyme encoded by the *blaZ* gene in *S. aureus* (5, 8). Originally, *S. aureus* Bla was classified into four variants (A, B, C, and D) based on serological properties and the hydrolysis kinetics against selected β-lactam antibiotics (9, 10). Single amino acid differences near the active site of Bla are responsible for the distinct enzymatic properties of these variants (11). Consequently, amino acid sequences have been used as genetic markers of Bla variants in various studies (5, 8, 12, 13). Among the four *blaZ* gene types, MSSA positive for the type A (*blaZ*_A_) gene is particularly associated with pronounced CIE, defined as an MIC of ≥16 µg/mL at high inoculum (5, 8, 9, 14). Threonine at position 128 (encoded by ACA) and serine at position 216 (encoded by AGC) of the *blaZ* gene, which characterizes type A Bla, have been suggested as markers for CIE. However, not all *blaZ*_A_ gene-positive MSSA strains exhibit CIE (13, 15, 16).

A previous study suggested that single nucleotide polymorphisms (SNPs) in the *blaZ*_A_ gene at codons 226 (proline [CCA] to serine [TCA]) and 229 (cysteine [TGT] to tyrosine [TAT]) in the amino acid sequence of *blaZ*_A_-positive MSSA are closely associated with CIE (15). However, it remains unclear whether these SNPs directly contribute to the CIE.

We constructed wild-type (WT) *blaZ_A_* gene expression plasmids (pBUS-Pcap-HC-blaZwt), inserting the PCR-amplified *blaZ_A_* gene of PNIDSA137, a *blaZ*_A_-positive MSSA clinical isolate with pronounced CIE, into the pBUS1-Pcap-HC plasmid (17). Then, we constructed three mutant *blaZ_A_* gene fragments with SNPs at codons 226 (blaZm649), 229 (blaZm659), and both 226 and 229 (blaZm649-659) and cloned them into the pBUS-Pcap-HC-blaZwt plasmid (Fig. S1). These plasmids were transformed into the *S. aureus* RN4220 strain, creating the following strains: WT-blaZ (RN4220-blaZwt), M226-blaZ (RN4220 with blaZm649), M229-blaZ (RN4220 with blaZm659), and MB-blaZ (RN4220 with blaZm649-659).

*blaZ_A_* gene expression levels and Bla protein production of the strains were measured by real-time PCR and Western blotting. To determine the inoculum effect of the experimental strains, the cefazolin MICs of the strains at high (HI, 5 × 10^7^ CFU/mL) and standard inocula (SI, 5 × 10^5^ CFU/mL) were measured and compared. Detailed materials and methods used in this study are described in the supplemental material.

**Fig 1:**
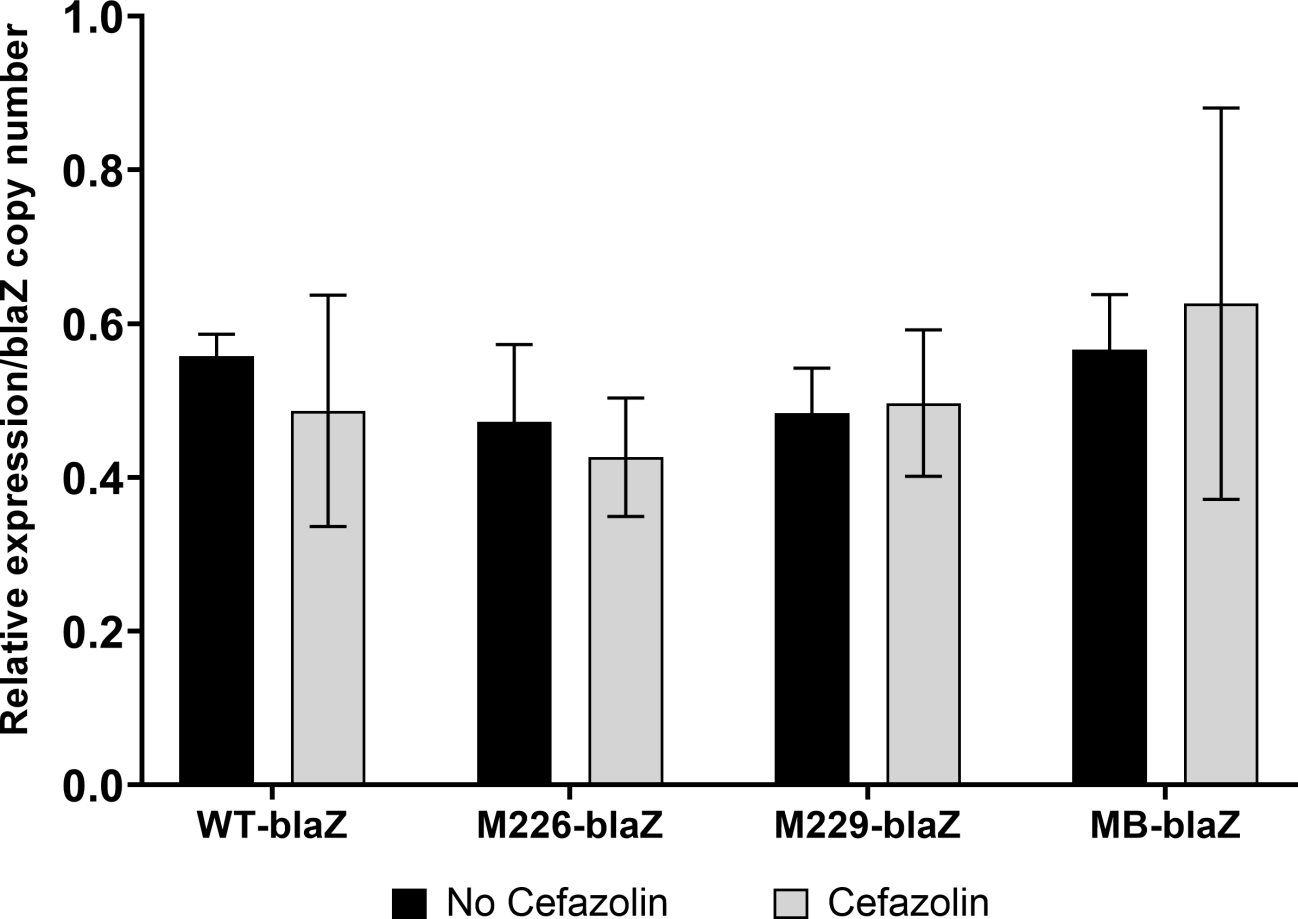
Comparison of relative *blaZ* gene expression, normalized to that of 16S rRNA, in WT and mutant *blaZ* gene-cloned *S. aureus* RN4220 using real-time quantitative polymerase chain reaction. *blaZ* gene expression did not significantly differ between cefazolin exposure and no cefazolin exposure groups after adjusting for the difference in copy numbers. (WT-blaZ [WT *blaZ_A_* gene], M226-blaZ [codon 226, sequence 649-mutated *blaZ_A_*], M229-blaZ [codon 229, sequence 659-mutated *blaZ_A_*], and MB-blaZ [both codons 226 and 229, sequence 649 and 659-mutated *blaZ_A_*].) The relative expression levels of blaZ were normalized to that of 16S rRNA.

**Fig 2:**
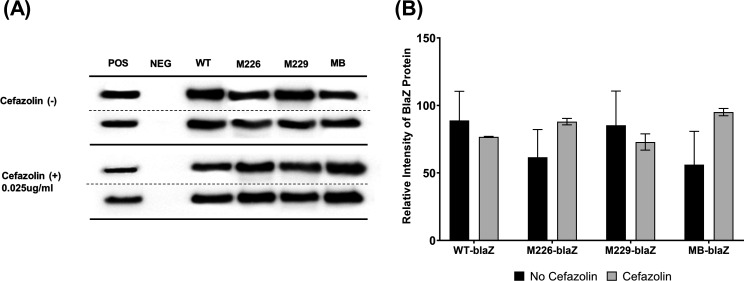
Western blotting of Bla protein in WT and mutant *blaZ* gene-cloned *S. aureus* RN4220; WT-blaZ (WT *blaZ*_A_ gene), M226-blaZ (codon 226-mutated *blaZ*_A_), M229-blaZ (codon 229-mutated *blaZ*_A_), and MB-blaZ (both codon 226 & 229-mutated *blaZ*_A_). Western blot findings (**A**) and plotting the relative intensity of Bla protein bands and Bla production did not significantly differ between WT and mutant *blaZ* (**B**).

The expression levels of the *blaZ*_A_ gene did not significantly differ between the cefazolin-exposed and unexposed conditions within each strain. When adjusting for the difference in copy numbers of the *blaZ*_A_-containing plasmids (Table 1), we found that the transformed *blaZ*_A_ gene in the WT-blaZ and mutant-blaZ strains were consistently expressed regardless of cefazolin exposure (Fig. 1), and the mutations did not affect the gene expression at the transcriptional level. The Western blotting results showed that Bla protein was produced in the WT and mutant *blaZ*_A_ gene-transformed strains (Fig. 2A). The relative intensity of Bla protein bands in the WT-blaZ strain was not significantly different from that in the M226-blaZ, M229-blaZ, and MB-blaZ strains (Fig. 2B).

**TABLE 1:** Characteristics of *S. aureus* RN4220 transformants and positions of single nucleotide polymorphisms of type A *blaZ* gene, Bla producing plasmid copy numbers, and cefazolin MICs^*a*^

Strain	Description	Site of mutation in type A *blaZ* gene^*b*^	PCN/cell (mean ± SD)	GM MIC of cefazolin at SI (μg/mL)	GM MIC of cefazolin at HI (μg/mL)
*S. aureus,* PNIDSA137	Clinical strain used for the *blaZ_A_* gene cloning template		–^*c*^	1	64
*S. aureus,* PNIDSA014	*blaZ* gene-negative clinical strain		–	0.25	0.5
*S. aureus,* WT-blaZ	*S. aureus,* RN4220 transformed with pBUS-Pcap-HC-blaZwt	649 **C**CA (proline 226), 659 T**G**T (cysteine 229)	3.90 ± 0.44	0.5	6.73
*S. aureus*, M226-blaZ	*S. aureus,* RN4220 transformed with pBUS-Pcap-HC-blaZm649 (codon 226 mutation)	649 **T**CA (serine 226), 659 T**G**T (cysteine 229)	2.74 ± 0.51	0.5	2.38
*S. aureus,* M229-blaZ	*S. aureus,* RN4220 transformed with pBUS-Pcap-HC-blaZm659 (codon 229 mutation)	649 **C**CA (proline 226), 659 T**A**T (tyrosine 229)	2.64 ± 0.51	0.5	6.73
*S. aureus*, MB-blaZ	*S. aureus,* RN4220 transformed with pBUS-Pcap-HC-blaZm649-659 (codons 226 and 229 mutation)	649 **T**CA (serine 226), 659 T**A**T (tyrosine 229)	2.37 ± 0.29	0.5	1.68

^*a*^ PCN, plasmid (*blaZ* gene harboring) copy number; GM MIC, geometric mean minimum inhibitory concentration; SI, standard inoculum; HI, high inoculum.

^*b*^ Bold and underlining indicate the single nucleotide mutation sites.

^*c*^ –, not applicable.

**Fig 3:**
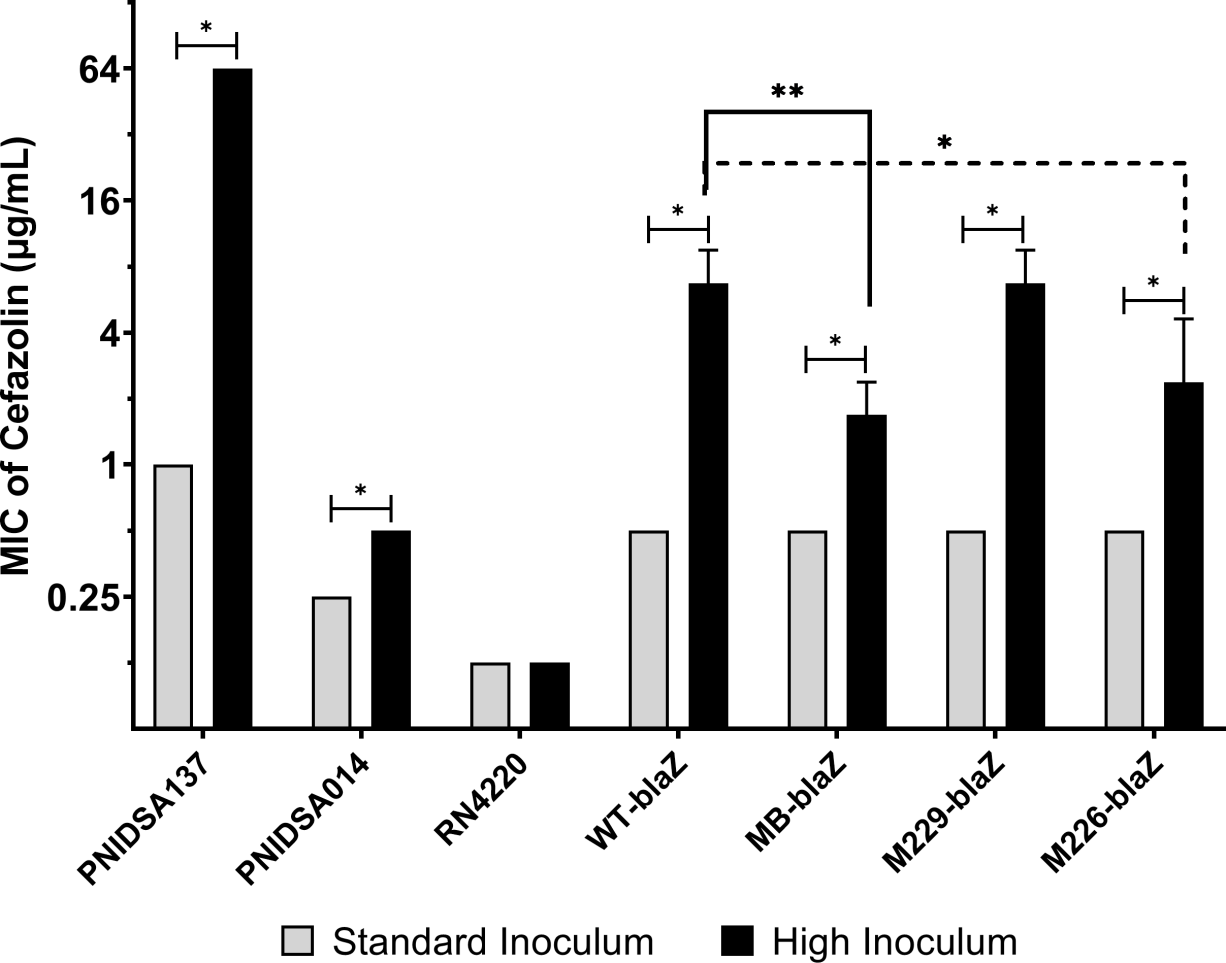
Comparison of cefazolin MIC between standard inoculum and high inoculum without (RN4220) and with (WT-blaZ, RN4220 harboring WT *blaZ_A_* gene) type A *blaZ* gene and comparison of HI cefazolin MICs WT *blaZ_A_-*containing versus mutant *blaZ_A_-*containing *S. aureus* (RN4220). (PNIDSA137, positive control strain of cefazolin inoculum effect; *P < 0.05 and **P < 0.005).

The cefazolin MIC in the WT-blaZ strain was 16-fold higher at HI (8 µg/mL) than at SI (0.5 µg/mL), whereas the untransformed RN4220 exhibited no increase in cefazolin MIC at HI compared with SI (Fig. 3).

To investigate CIE according to specific DNA mutations, the HI cefazolin MIC of the WT-blaZ strain was compared with that of the mutant *blaZ*_A_ gene-transformed RN4220 strains (M226-blaZ, M229-blaZ, and MB-blaZ). Compared with that in the WT-blaZ strain (6.73 µg/mL, 95% CI 3.88–11.68), the geometric mean MIC of HI cefazolin was significantly lower in the MB-blaZ (1.68 µg/mL, 95%CI 0.97–2.92, *P* = 0.002) and M226-blaZ (2.38 µg/mL, 95%CI 0.83–6.84, *P* = 0.015) strains but was not significantly different in the M229-blaZ strain (6.73 µg/mL, 95%CI 3.88–11.68, *P* > 0.999) (Fig. 3).

Our findings highlight the significant role of type A Bla in conferring CIE in *S. aureus*. Transformation of the *blaZ_A_* gene into the *S. aureus* RN4220 strain, which initially lacks CIE, resulted in the manifestation of CIE. This result confirms the association between the type A Bla and the CIE phenotype; however, it is conditional on the *blaZ_A_* sequence present.

In addition, our results emphasize that polymorphisms at specific sites in the *blaZ_A_* gene, particularly at codon 226, are crucial determinants for the presence of CIE. Interestingly, the double mutation at codons 226 and 229 induced more prominent CIE than the single mutation at codon 226, although the mutation at codon 229 alone does not appear to impact CIE. This finding suggests that the mutation at codon 229 augments the effect of the mutation at codon 226. While CIE can be influenced by various factors, our study shows that the SNP at codon 226 significantly reduces the HI cefazolin MIC in *S. aureus*. Mutations of single or multiple bases can alter the structure of the enzyme, potentially leading to changes in its activity or stability that result in either a loss- or gain-of-function (18). The mutation from proline (CCA) to serine (TCA) at base 659 (codon 226) of the *blaZ_A_* gene appears to be a loss-of-function mutation, reducing Bla activity. Interestingly, both the WT and mutant *blaZ_A_* genes induced a similar increase in MIC at SI, but the MIC at HI showed a greater than fourfold difference due to specific mutations in the *blaZ_A_* gene. This suggests that a significant portion of CIE is driven by changes in the enzymatic properties of Bla.

Particularly, our study demonstrates that single or multiple nucleotide sequence mutations can enhance the Bla activity of *S. aureus*. The impact of broad-spectrum Bla, such as extended-spectrum β-lactamases and carbapenemases in Gram-negative bacteria, has been significant (19, 20). Although the clinical impact of Bla in Gram-positive *S. aureus* has been less significant, continued antibiotic selection pressure could lead to the emergence of more potent Bla-producing strains.

In conclusion, the acquisition of the *blaZ_A_* gene contributes to the CIE in *S. aureus*, and the SNP at codon 226 in the *blaZ_A_* gene is one of the key factors in this effect. Our study underscores the need for judicious antibiotic use to mitigate the risk of developing resistant bacterial strains.

## SUPPLEMENTAL MATERIAL

Supplemental material aac.01106-24-s0001.docx Supplemental methods, Tables S1 and S2, and Fig. S1.

ASM does not own the copyrights to Supplemental Material that may be linked to, or accessed through, an article. The authors have granted ASM a non-exclusive, world-wide license to publish the Supplemental Material files. Please contact the corresponding author directly for reuse.
