# Supplementary material for: Polymorphisms in the type A blaZ gene as determinants of the cefazolin inoculum effect in Staphylococcus aureus
Source: Antimicrob Agents Chemother. 2024 Dec 10;69(1):e01106-24. doi: 10.1128/aac.01106-24 (PMC11784186; doi:10.1128/aac.01106-24)
Supplement: Supplemental material — Supplemental methods, Tables S1 and S2, and Fig. S1. [file aac.01106-24-s0001.docx]

**DETAILED EXPERIMENTAL METHODS**

**Bacterial strains and growth conditions**

We used *Escherichia coli* DH5α and *Staphylococcus aureus* RN4220 strains (Supplement table1). The strains were grown in Luria-Bertani (LB) broth with shaking at 220 rpm or on LB agar plates at 37°C. *E. coli* DH5α was transformed via the heat shock method, while *S. aureus* RN4220 was transformed via electroporation as previously described (1). RN4220 transformants were selected on agar plates containing 10 μg/mL tetracycline.

**DNA preparation, polymerase chain reaction (PCR), and sequencing**

Genomic DNA was extracted using spin column-based extraction kits (Qiagen, Hilden, Germany). The wild-type (WT) *blaZ*_A_ gene was amplified from the genomic DNA of PNIDSA137, a *blaZ*_A_-positive MSSA clinical isolate with pronounced CIE, using the following primers: 5’- TTATCATATGCTAGCAACTGTAATATCGGAGGGTTT-3’ and 5’- ATTCCTGCAGCTAGTGATATCAAAATTATACATGTCAACGA-3’. To ensure adequate lysis of *S. aureus,* the cells were incubated at 37°C for 15 min with 100 μg/mL lysostaphin (Sigma-Aldrich, St. Louis, MO, USA). PCR for plasmid construction was conducted using high-fidelity DNA polymerase (AccuPrime *Taq* DNA Polymerase, High Fidelity; Invitrogen, Carlsbad, CA, USA). The PCR products were purified using the Exo SAP-IT PCR Product Cleanup kit (Affymetrix Inc., Santa Clara, CA, USA).

**Construction of WT *blaZ_A_* expression plasmids and transformation**

We used the pBUS1-Pcap-HC plasmid expression system containing the *S. aureus* type 1 capsule gene 1A promoter to construct WT *blaZ_A_* gene expression plasmids, as performed by Schwendener et al. (2). The shuttle vector pBUS1-Pcap-HC was linearized with NheI, ligated to the purified PCR product of WT *blaZ*_A_ using the In-fusion HD Cloning kit (Takara Bio, Shiga, Japan), and transformed into *E. coli* DH5α cells. *E. coli* transformants were selected on LB plates containing 100 μg/mL tetracycline. The WT *blaZ_A_* gene expression plasmid pBUS-Pcap-HC-blaZwt was isolated from 2 mL cultures of individual colonies, and the DNA inserts were sequenced. The constructed plasmids were transformed into the *S. aureus* RN4220 strain via electroporation, generating the *S. aureus* WT-blaZ strain (RN4220 harboring pBUS-Pcap-HC-blaZwt).

**Construction of mutant *blaZ* expression plasmids and transformation**

We constructed three mutant *blaZ_A_* gene fragments with SNPs at codon 226 (blaZm649), 229 (blaZm659), and both 226 and 229 (blaZm649-659) and cloned them into the pBUS-Pcap-HC-blaZwt plasmid, which was digested with PacI and PstI (Fig. 1), to generate pBUS-Pcap-HC-blaZm649, pBUS-Pcap-HC-blaZm659, and pBUS-Pcap-HC-blaZm649-659, respectively (Table 2). These plasmids were transformed into the *S. aureus* RN4220 strain, creating the following strains: M226-blaZ (RN4220 with blaZm649), M229-blaZ (RN4220 with blaZm659), and MB-blaZ (RN4220 with blaZm649-659). The changes in base and amino acid sequences of mutant *blaZ*_A_ are presented in Table 3.

**Determination of plasmid copy number using real-time quantitative PCR (qPCR)**

Multiplex real-time qPCR was performed on an Applied Biosystems 7500 Real-Time PCR System (Thermo Fisher Scientific, Waltham, MA, USA) using primers specific for the chromosomal *S. aureus* nuclease gene (*nuc*) and the plasmid gene *tet*(L) (Table 4) (3). For standard curve preparation, pBUS1-Pcap-HC plasmids isolated from DH5α and genomic DNA from the RN4220 strain without plasmid were used. Separate qPCR standard curves for *nuc* and *tet*(L) were generated, measuring four-fold serial dilutions ranging from 20 ng (6,783,250 copies) to 76 fg (26 copies) and from 500 pg (80,926,176 copies) to 1.9 fg (309 copies), respectively. DNA copy number was calculated using the formula: DNA copy number = (g of DNA) x (6.022 x 10^23^ copies/mol)/(bp of DNA) x (665 g/mol/bp) (4). The chromosome size of RN4220 (2.67 Mb) was obtained from Nair et al. (5). DNA for plasmid copy number determination was obtained via crude lysis of four plasmid-containing RN4220 colonies. For qPCR, the lysates were diluted 1:10. Threshold cycle (*C_T_*) values of standards and samples were measured in triplicate with automatic threshold settings.

**Measurement of *blaZ_A_* gene expression using real-time PCR**

Total bacterial RNA was extracted from *S. aureus* WT-blaZ, M226-blaZ, M229-blaZ, and MB-blaZ strains grown with or without exposure to subinhibitory concentrations of cefazolin (1/20 x MIC). RNA extraction and cDNA synthesis were performed using the RNeasy® Mini Kit (Qiagen) and cDNA synthesis kit (DiaStar™ RT Kit, Solgent, Daejeon, Korea), respectively, according to the manufacturer’s instructions. Each PCR mixture had a total volume of 20 μL containing 10 μL of 2× Universal SYBR Green Master mix (Qiagen, Hilden, Germany), 5 pM forward and reverse primers (Table 4), and 1 μL of template. The relative expression levels of *blaZ* were normalized to that of 16S rRNA and calculated using the comparative CT method (2-^ΔΔCT^).

**Detection of Bla production by Western blotting**

Bla protein production in WT-blaZ, M226-blaZ, M229-blaZ, and MB-blaZ strains was examined using Western blotting. Cells were grown to the mid-logarithmic phase in Tryptic soy broth, harvested, washed, and resuspended in 50 mM Tris-HCl (pH 7.5). The cell suspensions were lysed using lysostaphin (100 μg/mL) and sonication. Total protein concentrations were determined using the Bradford assay (Bio-Rad Laboratories, Hercules, CA), and equal amounts of protein (20 μg) were separated via sodium dodecyl sulphate-polyacrylamide gel electrophoresis and transferred onto polyvinylidene difluoride membranes. The membranes were probed with a mouse anti-*S. aureus* penicillinase BlaZ antibody (USBiological Life Sciences, Salem, MA, USA) followed by a goat anti-mouse IgG secondary antibody (Enzo Life Sciences, Farmingdale, NY, USA). The protein bands were visualized using an ECL detection system (GE Healthcare, Little Chalfont, UK) and analyzed via densitometry using ImageJ software (NIH, Bethesda, MD, USA). Coomassie staining was used to control the total protein load and the blotting efficiency.

**Susceptibility testing to cefazolin at high and standard inocula**

The MICs of cefazolin for the constructed RN4220 strains were determined using a broth microdilution method in cation-adjusted Mueller-Hinton II broth (Becton, Dickinson and Company, Sparks, MD, USA), according to Clinical and Laboratory Standards Institute guidelines (6). Two inoculum sizes were tested: approximately 5 x 10^7^ CFU/ml (high inoculum, HI) and 5 x 10^5^ CFU/ml (standard inoculum, SI). The MIC was defined as the lowest concentration of cefazolin that completely inhibited visible bacterial growth after 24 h of incubation at 37°C. Each experiment was performed in triplicate.

**Statistical analysis**

For comparison of the results of real-time PCR, western blot assay, and MIC of the strains, statistical significance was determined using Student’s *t*-test or Mann-Whitney Wilcoxon test, as appropriate, with alpha < 0.05 using Prism for Windows (version 9.5.1; GraphPad Software, Inc., CA, USA).

**Supplement Table 1.** Strains and plasmids used in this study

| Strains and Plasmid | Description | Reference |
| --- | --- | --- |
| *E. coli*, DH5α | Transformation competent cell/ *E. coli* K-12 strain; *recA1* *endA1* *hsdR17* (r_K_^-^ m_K_^+^) | Thermo Fisher Scientific |
| *S. aureus*, RN4220 | Restriction-deficient cloning host | Lee JH et al.(7) |
| *S. aureus*, TX0117 | Positive control Strain of Cefazolin Inoculum effect | Nannini EC et al.(8) |
| *S. aureus*, PNIDSA014 | Negative control Strain of Cefazolin Inoculum effect (HI MIC = 0.5 μg/mL and SI MIC = 0.25 μg/mL) | This study |
| *S. aureus* PNIDSA137 | Cefazolin Inoculum Effect (HI MIC 64 μg/mL and SI MIC = 1 μg/mL) and Type A Bla positive strain used for *blaZ_A_* gene cloning template | This study |
| Plasmid, pBUS1-Pcap-HC | *E. coli*-*S. aureus* shuttle vector plasmid, containing the *S. aureus* type 1 capsule gene 1A promoter | Schwendener S. et al.(2) |
| Plasmid, pBUS-Pcap-HC-blaZwt | Wild-type *blaZ_A_* gene inserted pBUS1-HC | This study |
| Plasmid, pBUS-Pcap-HC-blaZm649 | 649 (C → T) single-base mutated *blaZ_A_* gene inserted pBUS1-HC | This study |
| Plasmid, pBUS-Pcap-HC-blaZm659, | 659 (G → A) single-base mutated *blaZ_A_* gene inserted pBUS1-HC | This study |
| Plasmid, pBUS-Pcap-HC-blaZm649-659 | 649 and 659 mutated *blaZ_A_* gene inserted pBUS1-HC | This study |
| *S. aureus*, WT-blaZ | *S. aureus*, RN4220 transformed with pBUS-Pcap-HC-blaZwt, Cefazolin Inoculum Effect Positive phenotype | This study |

**Supplement Table 2.** PCR primers used in this study

| Name | Sequences | size (bp) | Reference |
| --- | --- | --- | --- |
| Primer for cloning *blaZ* insert (High fidelity PCR) | | | |
| *blaZ*-F | TTATCATATGCTAGCAACTGTAATATCGGAGGGTTT | 1054 | This study |
| *blaZ*-R | ATTCCTGCAGCTAGTGATATCAAAATTATACATGTCAACGA |  |  |
| Primer for blaZ gene sequencing | | | |
| *blaZ*-F | CAAAGATGATATAGTTGCTTATTC | 355 | Nannini EC *et al.* 2009 (9) |
| *blaZ*-R | CATATGTTATTGCTTGCACCAC |  |  |
| Primer for plasmid copy number counting | | | |
| *nuc*-F | AAA GCG ATT GAT GGT GAT ACG GTT | 93 | Wang HY et al. 2014 (3) |
| *nuc*-R | TGC TTT GTT TCA GGT GTA TCA ACC A |  |  |
| *tetL*-F | GGC TTT CGT TCA CCA AAA CAG T | 96 | Schwendener et al. 2015 (2) |
| *tetL*-R | TGG TAA AGT TAA GCA AAC TCA TTC CA |  |  |
| Primer for real-time RT qPCR | | | |
| 16S rRNA-F | TCC GGA ATT ATT GGG CGT AA | 119 | Goldstein F et al. 2007 (10) |
| 16S rRNA-R | CCA CTT TCC TCT TCT GCA CT |  |  |
| *blaZ*-F | TCC TAA GGG CCA ATC TGA ACC | 105 | Chovanová R et al. 2016 (11) |
| *blaZ*-R | ACA CTC TTG GCG GTT TCA CT |  |  |

**Supplement Figure 1.** The schematic diagram for the construction of β-lactamase-producing *E. coli*-*S. aureus* shuttle vector. **(A)** The *blaZ*_A_ gene from *S. aureus* PNIDSA137 was amplified and inserted at the NheI (78) site within the multiple cloning sites of pPUS1-Pcap-HC. **(B)** In pBUS1-pCap-HC-blaZwt, WT *blaZ_A_* gene fragment was placed with mutant *blaZ_A_* gene fragments with base substitutions at codons 226 and/or 229 between the PacI (738) and PstI (1045) restriction sites.


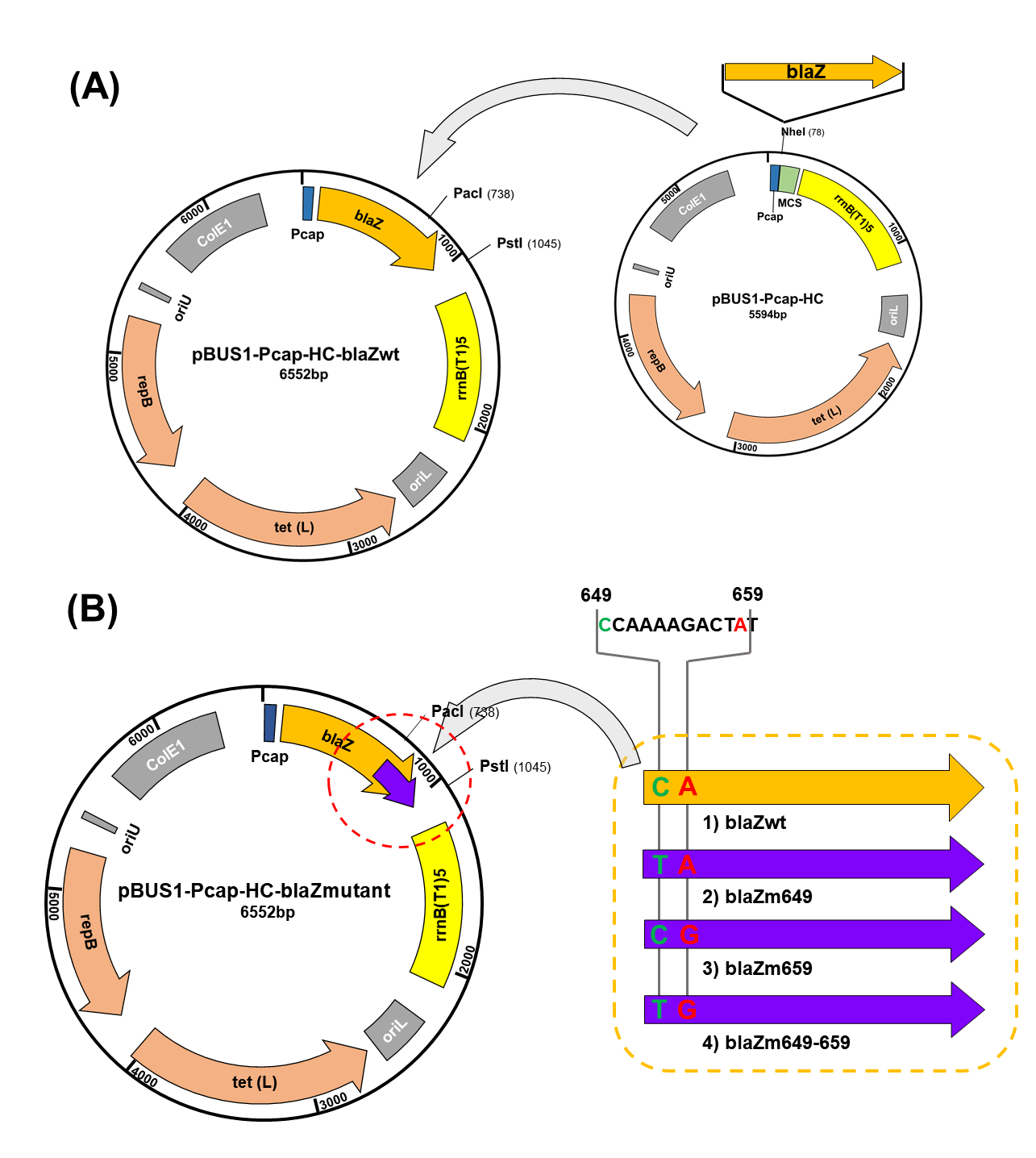


References

1. Schenk S, Laddaga RA. 1992. Improved method for electroporation of Staphylococcus aureus. FEMS Microbiol Lett 73:133-8.

2. Schwendener S, Perreten V. 2015. New shuttle vector-based expression system to generate polyhistidine-tagged fusion proteins in Staphylococcus aureus and Escherichia coli. Appl Environ Microbiol 81:3243-54.

3. Wang HY, Kim S, Kim J, Park SD, Uh Y, Lee H. 2014. Multiplex real-time PCR assay for rapid detection of methicillin-resistant staphylococci directly from positive blood cultures. J Clin Microbiol 52:1911-20.

4. Li Z, Hansen JL, Liu Y, Zemetra RS, Berger PH. 2004. Using real-time PCR to determine transgene copy number in wheat. Plant Molecular Biology Reporter 22:179-188.

5. Nair D, Memmi G, Hernandez D, Bard J, Beaume M, Gill S, Francois P, Cheung AL. 2011. Whole-genome sequencing of Staphylococcus aureus strain RN4220, a key laboratory strain used in virulence research, identifies mutations that affect not only virulence factors but also the fitness of the strain. J Bacteriol 193:2332-5.

6. Institute CaLS. 2008. Methods for dilution antimicrobial susceptibility tests for bacteria that grow aerobically; approved standard, 7 ed. Clinical and Laboratory Standards Institute, Wayne, PA.

7. Lee J-H, Kim N-H, Winstel V, Kurokawa K, Larsen J, An J-H, Khan A, Seong M-Y, Lee Min J, Andersen Paal S, Peschel A, Lee Bok L. 2015. Surface Glycopolymers Are Crucial for In Vitro Anti-Wall Teichoic Acid IgG-Mediated Complement Activation and Opsonophagocytosis of Staphylococcus aureus. Infection and Immunity 83:4247-4255.

8. Nannini EC, Singh KV, Murray BE. 2003. Relapse of type A beta-lactamase-producing *Staphylococcus aureus* native valve endocarditis during cefazolin therapy: revisiting the issue. Clin Infect Dis 37:1194-8.

9. Nannini EC, Stryjewski ME, Singh KV, Bourgogne A, Rude TH, Corey GR, Fowler VG, Jr., Murray BE. 2009. Inoculum effect with cefazolin among clinical isolates of methicillin-susceptible Staphylococcus aureus: frequency and possible cause of cefazolin treatment failure. Antimicrob Agents Chemother 53:3437-41.

10. Goldstein F, Perutka J, Cuirolo A, Plata K, Faccone D, Morris J, Sournia A, Kitzis MD, Ly A, Archer G, Rosato AE. 2007. Identification and phenotypic characterization of a beta-lactam-dependent, methicillin-resistant Staphylococcus aureus strain. Antimicrob Agents Chemother 51:2514-22.

11. Chovanová R, Mikulášová M, Vaverková Š. 2016. Modulation of mecA Gene Expression by Essential Oil from Salvia sclarea and Synergism with Oxacillin in Methicillin Resistant Staphylococcus epidermidis Carrying Different Types of Staphylococcal Chromosomal Cassette mec. Int J Microbiol 2016:6475837.
